# Jog with your dog: Dog owner exercise routines predict dog exercise routines and perception of ideal body weight

**DOI:** 10.1371/journal.pone.0272299

**Published:** 2022-08-24

**Authors:** Sydney Banton, Michael von Massow, Júlia G. Pezzali, Adronie Verbrugghe, Anna K. Shoveller

**Affiliations:** 1 Department of Animal Biosciences, Ontario Agricultural College, University of Guelph, Guelph, Ontario, Canada; 2 Department of Food, Agricultural & Resource Economics, Ontario Agricultural College, University of Guelph, Guelph, Ontario, Canada; 3 Department of Clinical Studies, Ontario Veterinary College, University of Guelph, Guelph, Ontario, Canada; University of Lincoln - Brayford Campus: University of Lincoln, UNITED KINGDOM

## Abstract

Canine obesity is becoming an increasingly prevalent concern among companion animal veterinarians and professionals alike. A number of sociodemographic, dietary, and exercise related variables have been shown to be predictive of a dog’s bodyweight, however, all previous surveys designed to address these variables have been focussed on only one area of the world at a time. The objective of this survey was to investigate how an owner’s exercise routine influences their dog’s exercise routine and which of the owner’s dietary and exercise habits influence their perception of their dog’s body weight. The survey included respondents across France, Germany, the United Kingdom, Canada and the United States. The survey was distributed online via Qualtrics (Qualtrics XM, Utah, USA) and a total of 3,298 responses were collected, equally distributed across country and between sexes. Comparison of column proportions and multinomial logistic regression were performed in SPSS Statistics (Version 26, IBM Corp, North Castle, New York, USA). Respondents from Germany were more likely to exercise their dog for a longer amount of time, rank the importance of exercise as extremely important, report that their dog is an ideal body weight, and were less likely to report that someone (including a veterinarian) had told them their dog was overweight. Results from linear regression revealed that those who had been told their dog was overweight, those who restrict their dog’s food intake to control weight, those who select a weight control diet and those who give their dog more other foods (treats, table scraps, fruits/vegetables) on a daily basis were all less likely to believe that their dog is an ideal body weight. In contrast, only those who reported doing more vigorous exercise themselves or those who reported that their dog performs vigorous exercise were more likely to believe that their dog is an ideal body weight. The results highlight owner’s perceptions of healthy weight and the role of nutrition and exercise. Owner’s intentions and attitudes towards the value of exercise and promoting an ideal body weight in their dog should be explored, but may require a One Health approach to ensure successful weight management among both dogs and their owners.

## Introduction

Obesity in dogs has become an increasingly prevalent health concern across the globe [1]. Estimates of overweight and obese dogs in the global canine population range from 34–41% [2, 3]. Overweight or obese animals are at an increased risk of developing several diseases, including: orthopedic, skin, respiratory, cardiac and renal diseases, diabetes mellitus and a reduced life span [reviewed in 4]. Obesity is a multifactorial disease. There are many risk factors associated with canine obesity. Some breeds tend to be predisposed to becoming overweight or obese, such as the Cocker Spaniel, Labrador Retriever and Beagle [5]. In addition, gonadectomy can increase the risk of obesity in dogs as well [6]. However, the main cause of obesity is a positive energy balance, mostly caused by excessive energy intake, due to overfeeding and/or lack of exercise, both of which are preventable [7]. While there are many guidelines for owners on how to feed their dogs, including the back of every pet food label, there are limited resources backed by scientific evidence for how to exercise your dog, making it more difficult for an owner to know how much exercise is enough for their dog. The United States Department of Agriculture recommends a minimum of 30 minutes of exercise per day, based on kennel-housed dogs [8]. The American Animal Hospital Association provides guidelines for exercising an obese dog by starting with a 5-minute walk, three times a day and increasing gradually to a total of 30–45 minutes of walking per day, but this can depend on size, age and health status of the dog [9]. Dietary and exercise regimens of companion animals are entirely determined by their owners, which may be closely related to their own dietary and exercise routine. Thus, understanding how different feeding and behavioural practices of dog owners influence a dog’s body weight may provide insights into how to better treat canine obesity.

Several researchers have attempted to understand those practices via survey. For example, in a survey conducted in Germany, owners of obese dogs were more likely to allow their dog to sleep in their bed, spent more time talking to their dog, rated exercise as less important for their dog, and, fed more frequent meals, treats and table scraps [10]. This suggests that owners who “over-humanize” their dog or use food as a positive interaction with their dog are more likely to have an obese dog. In addition, owners of obese dogs were more likely to be obese themselves and care less about their own health, suggesting that owner’s own health practices can influence their dog’s body weight [10]. Other surveys from various countries in Europe echo these findings, with the most common findings being that obese dogs receive less exercise and more human foods/table scraps [11, 12]. Furthermore, human dietary patterns such as vegan, high protein, organic, to name a few, have become increasingly popular in the pet food industry and an owner’s dietary habits can influence how they choose to feed their dog, which in turn, can influence their dog’s body weight [13].

Despite being a global health issue, research on owner attitudes towards companion animal obesity largely has focussed on the area of the world the research group is from. In addition, all of these previous surveys use some form of body condition scoring (BCS) or confirmation from a veterinarian in order to verify if the dog is overweight. However, part of the problem may be that an owner’s perception of their dog’s body weight does not align with what is considered overweight by a veterinarian [14]. Therefore, it is important to understand what factors contribute to an owner’s perception of their dog’s body weight without comparison to the veterinarian’s assessment, as it is perception that underlies owner behaviour [15].

Therefore, the objective of the current study was to take a comparative approach and investigate an owner’s *perception* of their dog’s body weight in dog owners from France, Germany, the United Kingdom (UK), Canada and the United States of America (USA). Furthermore, our research group sought to explore how an owner’s exercise routine may affect their dog’s exercise routine and which dietary and exercise variables predict the owner’s perception of their dog’s body weight. We hypothesized that an owner’s own exercise routine would influence their dog’s exercise routine, that such routines would be different between countries and that an owner’s dietary and exercise routine would influence their perception of their dog’s body weight.

## Materials and methods

### Survey

The complete “Pet Food Consumer Habits” survey and methodology can be found in [13]. Briefly, the survey consisted of 69 questions related to owner and dog demographics, diet and exercise regimens, allergies in dogs, body weight of dogs and pet food purchasing habits. The focus of the current study was to investigate the relationship between owner and dog dietary and exercise routines in relation to perception of ideal body weight in dogs. The questions related to owner exercise regimen were adapted from the International Physical Activity Questionnaire (IPAQ) [16]. Four multiple choice questions were used to assess how often the respondent performs vigorous exercise or moderate exercise and how often the respondent spends walking or sitting in a regular week. Vigorous exercise is defined in the IPAQ as physical activity that takes hard physical effort and makes you breathe much harder than normal, which can include things such as heavy lifting, aerobics and fast cycling. Moderate exercise is defined in the IPAQ as physical activity that takes moderate physical effort and makes you breathe somewhat harder than normal, which can include such things as lifting light loads, doubles tennis and cycling at a regular pace. In addition, the survey asked if the respondent does moderate exercise with their dog (defined as hiking, jogging, rollerblading or cycling with their dog) and how their exercise regimen has changed since getting a dog in the form of a Likert scale from less active (0) to more active (10). The owner was asked which dietary routines they follow with more than 20 options to select. The respondent was asked two ‘Yes/No’ questions about their dog’s body weight; if they believe their dog is an ideal body weight and if anyone (including a veterinarian) has ever told them their dog is overweight. Two multiple choice questions assessed the dogs exercise regimen; how much physical activity the dog performs (in minutes) and what type of physical activity the dog performs. The type of activity was further split into two categories for analysis; vigorous exercise in the dog was defined as running, playing ball, swimming or agility and moderate exercise was defined as walking, hiking, off leash walking or visiting the dog park. Several multiple choice questions assessed the dog’s dietary regimen including; how often the dog is fed, what other foods the dog receives, a series of ‘True/False’ questions about how much food the dog is fed and what type of food the owner looks for. Finally, the respondents were asked to rate the importance of exercise in terms of their dog’s overall health on a Likert scale from 0 (not important) to 10 (extremely important).

### Data collection

Data were collected as part of a previous publication [13] and was approved by the University of Guelph’s Research Ethics Board (REB 19-12-026). The survey was distributed online via Qualtrics (Qualtrics XM, Utah, USA) to respondents in Germany, the UK, France, the USA and Canada in June of 2020. These countries were selected to represent the larger pet food consuming countries in North America and Europe and the survey was translated to German and French. Qualtrics was responsible for recruiting all participants randomly via email and a quota was set for each country and sex in each country. Briefly, written consent was obtained from each respondent before completing the survey and all data were analyzed anonymously. In order to be eligible to complete the survey, respondents could not be a veterinarian, dog breeder or work in the pet food industry. Respondents had to be over the age of 18 years, own at least one dog that is consuming a non-prescription dry dog food (kibble) and be the primary person responsible for selecting their dog’s kibble. Only those feeding a commercial kibble diet were included in order to reduce variability in responses of those who feed non-traditional diets and because the majority (>80%) of dog owner’s in the United States feed a kibble diet [17].

### Statistical analysis

Survey data were analyzed in SPSS Statistics (Version 26, IBM Corp, North Castle, New York, USA). Frequency data were analyzed from all countries together and a two-sided test of equality for column proportions was done to compare proportions between variables. Pairwise comparisons were adjusted using the Bonferroni correction. Multinomial logistic regression was performed in order to determine which variables were significant in predicting a dog owner’s perception of their dog being an ideal body weight. After initial review of the data, it was found that more than half of the respondents from the USA were over the age of 65 years. Therefore, the interaction term of being from the USA and being 65 years or older was included in the regression model to determine if the skewed age data had an effect on the outcome of the model. The dependent variable was the binary outcome: selected ‘yes’ or ‘no’ to the question, “do you believe your dog is an ideal body weight?” Statistical significance was declared at P≤0.05.

## Results

### Demographics

A total of 3,298 complete responses were used for data analysis. Responses were equally distributed between countries (Canada = 657, USA = 657, Germany = 656, France = 661, UK = 667) and sexes (Male = 1,648, Female = 1,650). Results pertinent to the current discussion about body weight and exercise are presented below and were not reported in the previous publication. Other results, including human and dog demographics and diet, and pet food purchasing habits can be found in [13].

### Exercise

**Fig 1:**
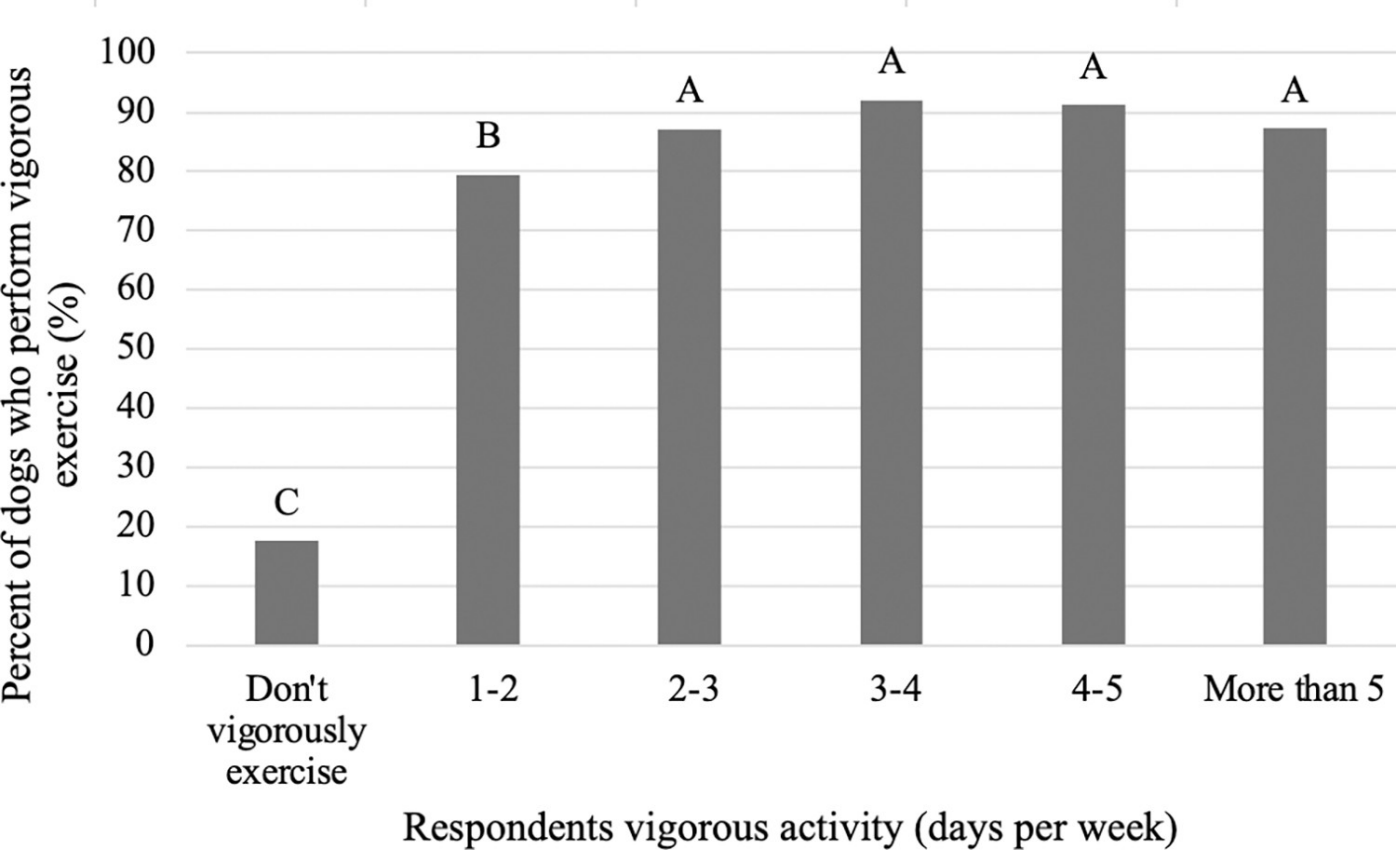
Percent of dogs that perform vigorous exercise among respondents who do or do not perform vigorous exercise (n = 3,298). Different letters are significantly different at P<0.05.

**Fig 2:**
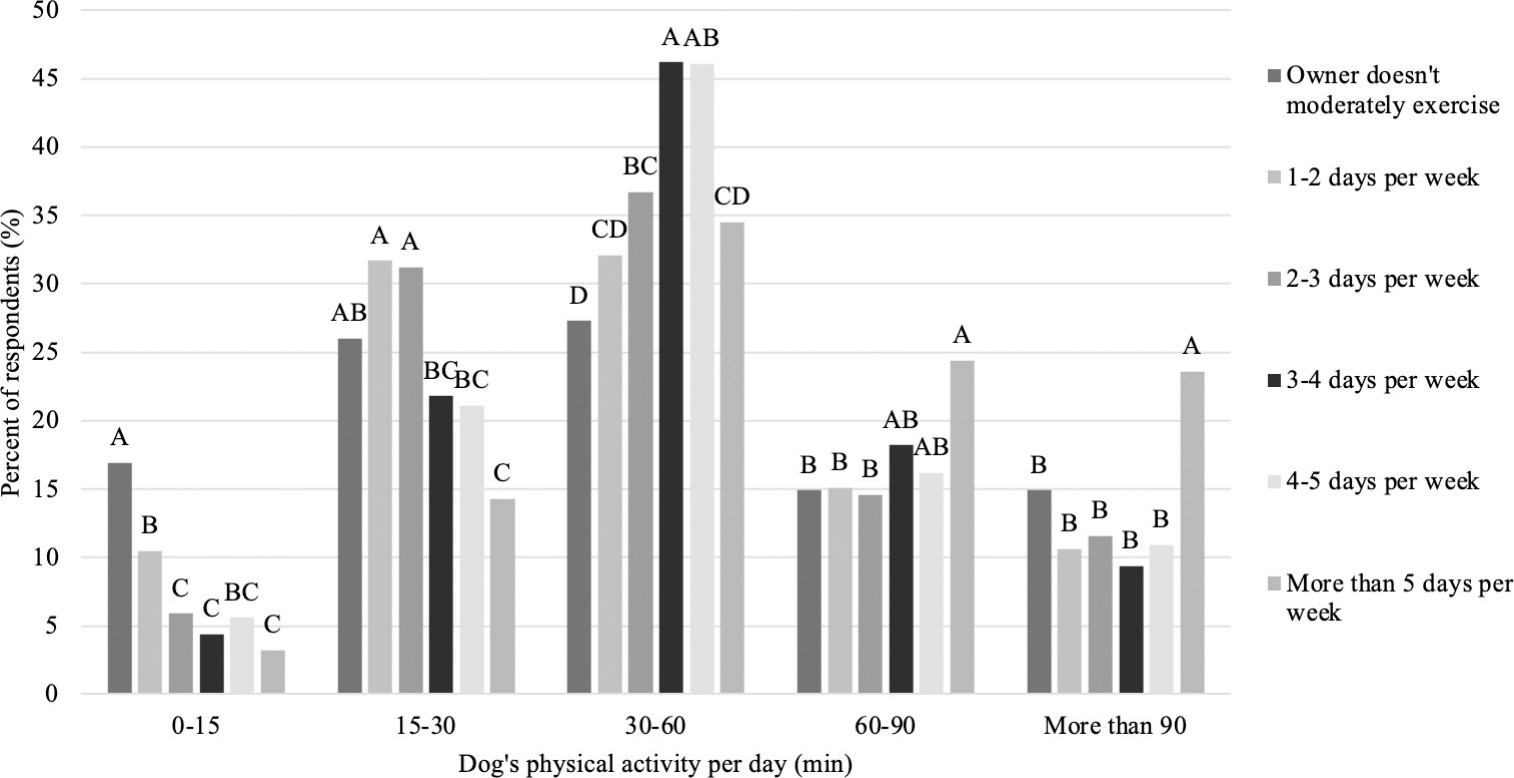
Difference in respondents time spent exercising their dog among time spent performing moderate exercise themselves (n = 3,298). Different letters are significantly different at P<0.05.

The frequency data below is reported for all countries combined. Of the four questions used to assess the humans’ exercise regimen, a majority of respondents reported vigorously exercising (69.0%) or moderately exercising (75.2%) for more than 15 minutes at least one day per week. The most common response was 1–2 days per week for both vigorous exercise (20.6%) and moderate exercise (24.6%). Similarly, a majority of respondents reported walking (92.2%) or sitting (97.2%) for more than 15 minutes at least one day per week. However, the most common response was more than 5 days per week for both walking (39.6%) and sitting (64.5%). Respondents who reported that they do not perform more than 15 minutes of vigorous exercise in a regular week were less likely to report that their dog performs vigorous exercise compared to those who reported performing vigorous exercise at least one day a week or more (Fig 1; P<0.001). However, there was no difference in the percent of dogs who perform vigorous exercise among owners who vigorously exercise for 2–3, 3–4, 4–5 or more than 5 days per week (P>0.05). Results suggest a similar pattern, although not overly strong, between those who reported doing moderate physical exercise and the amount of time their dog spends exercising (Fig 2). In general, dogs that received less exercise (0–15 and 15–30 min) had owners who were more likely to report spending less time performing moderate exercise themselves. In comparison, dogs that received more exercise (30–60, 60–90 and more than 90 minutes) had owners who were more likely to report spending more time performing moderate exercise themselves (Fig 2; P<0.001).

**Fig 3:**
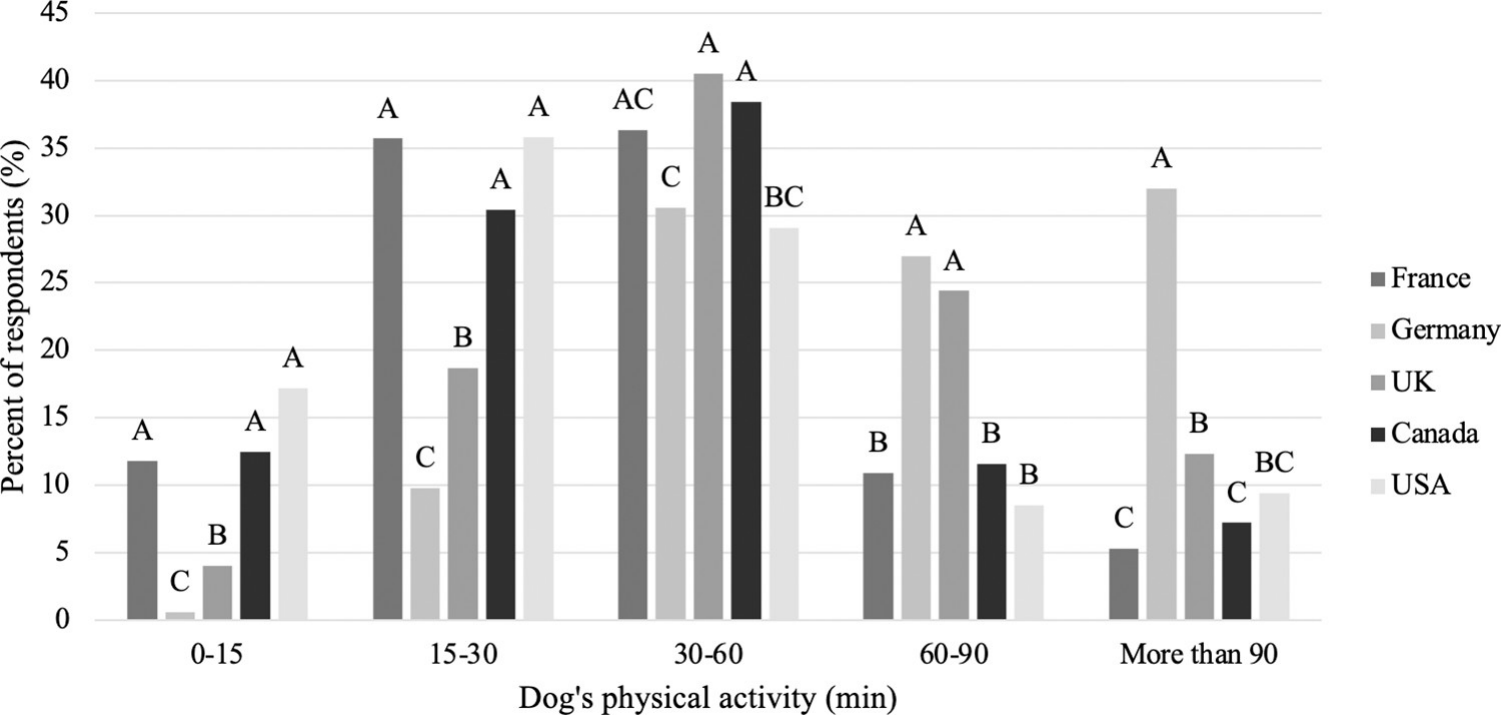
Difference in respondents time spent exercising their dog among countries (n = 3,298). Different letters are significantly different at P<0.05.

In terms of the dogs’ physical activity, the most common amount of time spent exercising was 30–60 minutes per day (35.0%), followed by 15–30 minutes (26.1%), 60–90 minutes (16.5%), more than 90 minutes (13.2%) and 0–15 minutes (9.2%). When this variable was looked at across countries, a number of differences were observed (Fig 3). Respondents from France (11.8 and 35.7%), the USA (17.2 and 35.8%) and Canada (12.5 and 30.4%) were all more likely to exercise their dog for a shorter amount of time (0–15 and 15–30 minutes, respectively) compared to the UK (4.0 and 18.7%) and Germany (0.6 and 9.8%; P<0.001). Respondents from Germany were more likely to exercise their dog for longer (27.0% for 60–90 minutes and 32% for more than 90 minutes) compared to the other countries (Fig 3; P<0.001). The most common response selected among German respondents was more than 90 minutes of exercise per day and those from Germany (53.2%) were more likely to rate the importance of exercise as 10 (extremely important) compared to all other countries (UK: 45.0%, USA: 38.1%, Canada: 32.4% and France: 19.7%; P<0.001).

The most common type of exercise for dogs was walking (89.3%), followed by playing ball (45.1%), running (43.3%), off leash walking (37.3%), hiking (14.8%), dog park (12.3%), swimming (7.8%) and agility (4.9%).

A majority (65.8%) of respondents reported doing moderate exercise with their dog regularly. Of those who responded ‘yes’, 48.5% reported doing this for 30–60 minutes per day, 38.9% reported doing this for 0–30 minutes per day and 12.5% reported doing this for more than 60 minutes per day. When asked how their exercise regimen has changed after getting a dog, a majority (71%) of respondents assigned a score of 6–10, more active, 26.8% of respondents assigned a score of 5, no change, and 2.2% assigned a score of 0–4, less active.

### Body weight

**Fig 4:**
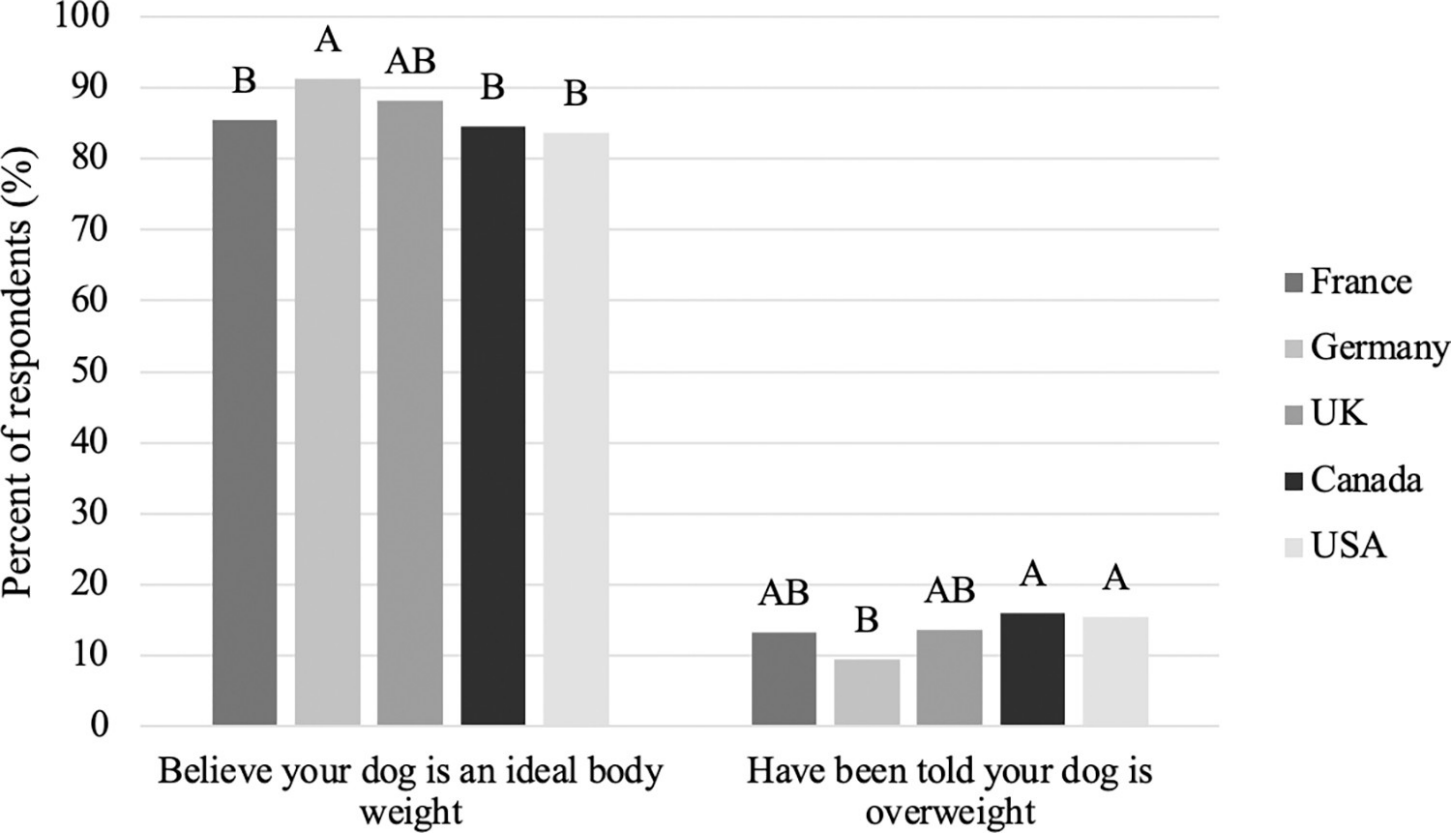
Percent of respondents among countries that reported believing their dog is an ideal body weight or have been told that their dog is overweight (n = 3,298). Different letters are significantly different at P<0.05.

When asked about their dog’s body weight, only 13.3% of respondents reported that they do not believe their dog is an ideal body weight and similarly, 13.6% of respondents reported that someone, including a veterinarian, has told them that their dog is overweight. Those who reported that someone has told them their dog is overweight were more likely to also report restricting their dog’s food intake to control weight (P<0.001) and giving their dog two or more other foods (treats, table scraps, fruits and veggies or other) on a daily basis (P = 0.004). When comparing countries, respondents from Germany (91.3%) were more likely to believe that their dog is an ideal body weight compared to those from France (85.5%), Canada (84.6%) and the USA (83.7%; Fig 4; P<0.001). Similarly, respondents from Germany (9.5%) were less likely to report that someone has told them their dog is overweight compared to those from Canada (16.0%) and the USA (15.4%; Fig 4; P = 0.006).

**Fig 5:**
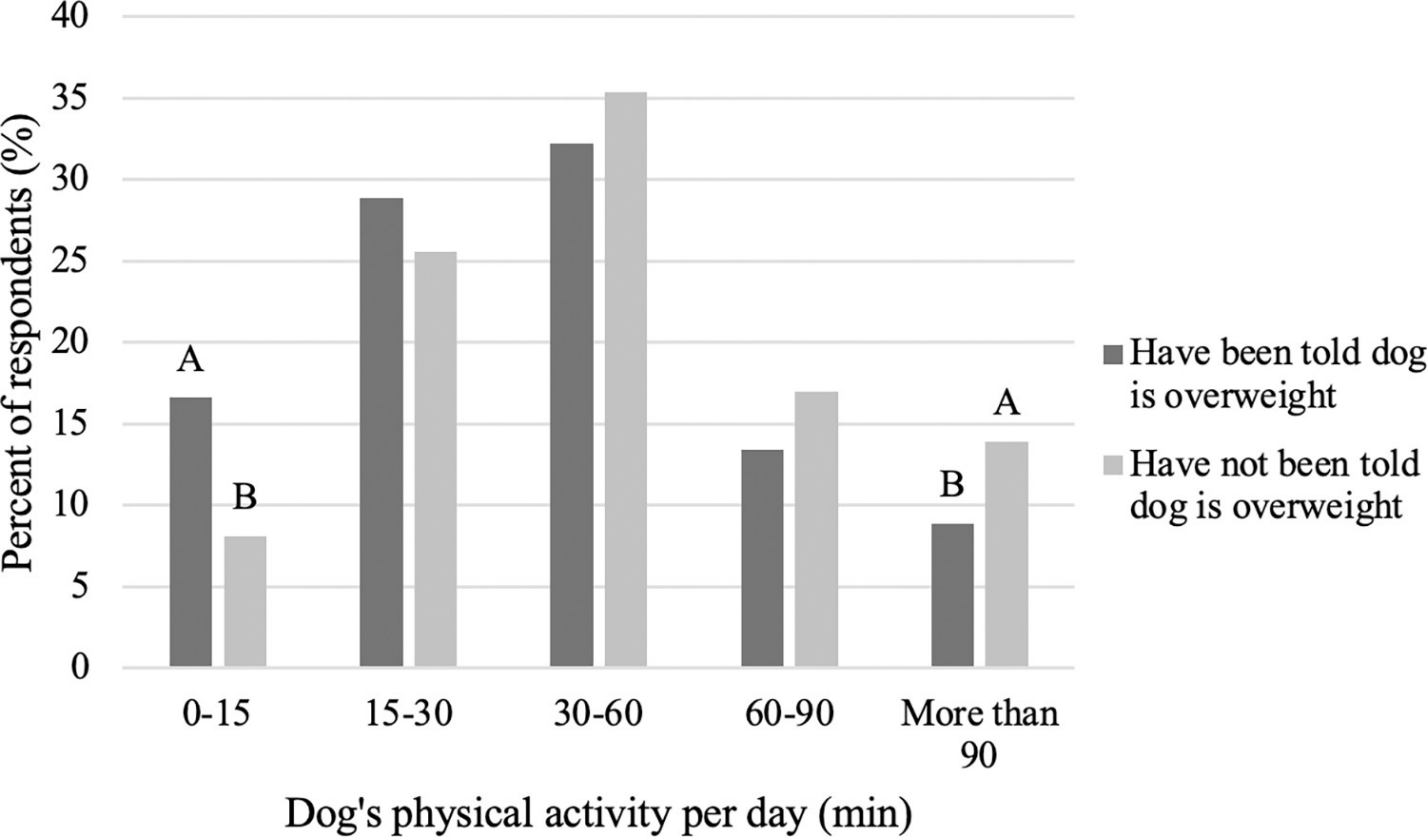
Difference in time spent exercising their dog among those who have or have not been told their dog is overweight (n = 3,298). Different letters are significantly different at P<0.05.

**Fig 6:**
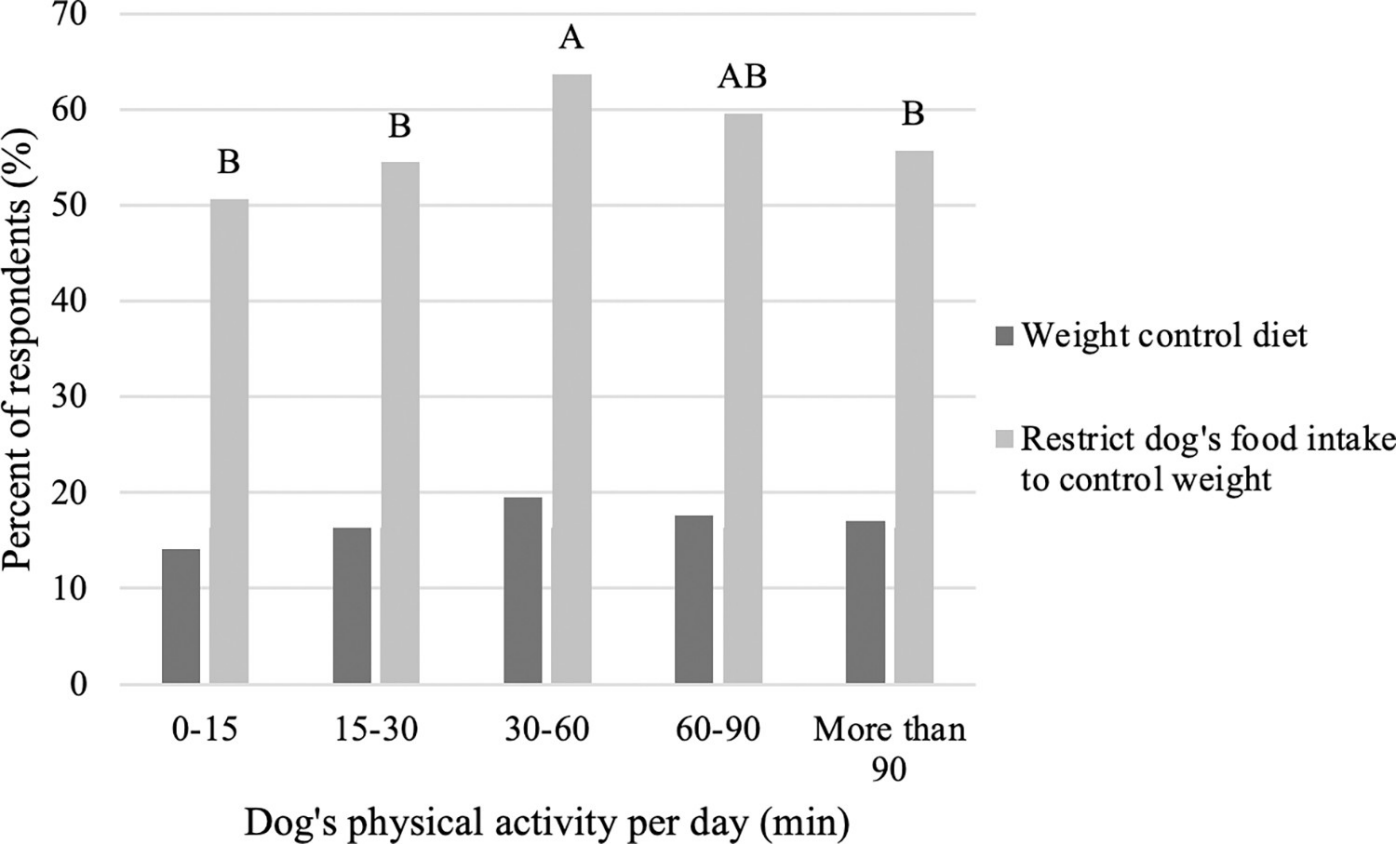
Difference in time spent exercising their dog among those who select a weight control diet for their dog or restrict their dog’s food intake to control weight (n = 3,298). Different letters are significantly different at P<0.05.

When comparing the influence of someone else (including a veterinarian) telling you that your dog is overweight and amount of time spent exercising, a pattern emerges. That is, those who have been told their dog is overweight are more likely to spend only 0–15 minutes exercising and less likely to spend more than 90 minutes exercising their dog compared to those that have not been told their dog is overweight (Fig 5; P<0.001). Similarly, among those who selected a weight control diet for their dog, there was no difference in amount of exercise their dog receives (P>0.05) and among those who restrict their dog’s food intake to control weight, only 30–60 minutes of exercise was more frequently selected compared to 0–15, 15–30 and more than 90 minutes of exercise for their dog (Fig 6).

### Multinomial logistic regression

Preliminary analysis suggested that owners of dogs who were 0–2 and 2–5 years of age were always more likely to believe that their dog was an ideal body weight. Therefore, in order to assess the variables that were predictive of an owner perceiving their dog as not an ideal body weight, only dogs who were 5 years of age or older were included in the regression model (n = 1,877). Variables that could potentially contribute to the dog becoming overweight (i.e. over feeding treats) and variables that could be a consequence of believing your dog is overweight (i.e. selecting a weight control diet) were added to the model to understand multiple correlations.

The regression model (χ^2^(37) = 699.82, P<0.001, Table 1) with the highest McFadden Pseudo R-Square (0.40) revealed that those who reported that they restrict their dog’s food intake to control their dog’s body weight (P = 0.011), those who look for a weight control pet food (P = 0.001), those who give one (P = 0.007) or two or more (P = 0.001) other food items to their dog on a daily basis and those who responded ‘yes’ to the question, “has anyone (including your veterinarian) ever told you that your dog is overweight?” (P<0.001) were all less likely to believe that their dog is an ideal body weight. In fact, believing that your dog is an ideal body weight was negatively correlated with being told that your dog is overweight (r = -0.61, P<0.01) and the addition of this variable to the model increased the Pseudo R-Square value by more than double. On the other hand, those who reported spending 4–5 (P<0.001) or 5 or more days (P = 0.004) per week vigorously exercising were 3.5 and 2.8 times more likely to believe their dog is an ideal body weight, respectively, compared to those who do not vigorously exercise. Similarly, those who reported that their dog performs vigorous exercise (running, playing ball, swimming and agility) were 1.8 times more likely to believe their dog is an ideal body weight (P<0.001). Those who reported that this was their first dog were 2 times more likely to believe that their dog is an ideal body weight (P = 0.002). When an interaction term of this being your first dog and the time spent exercising your dog was included in the model, it revealed that the more time spent exercising their dog, the more likely the respondent was to believe their dog is an ideal body weight, if this was their first dog (calculated OR: 0–15 minutes = 2.6, 15–30 minutes = 3.3, 30–60 minutes = 3.8). The respondents age, sex, country of residence and own dietary routines were not significant in explaining the variation in perception of their dog’s body weight.

**Table 1:** Multinomial logistic regression parameter estimates.

Variable	β^1^	Std. Error	P-Value	OR^2^	95% CI^3^	
					Lower Bound	Upper Bound
**Age**						
25–34 years	0.058	0.628	0.926	1.060	0.310	3.626
35–44 years	0.249	0.609	0.682	1.283	0.389	4.229
45–54 years	0.183	0.605	0.763	1.200	0.367	3.929
55–64 years	-0.148	0.604	0.807	0.863	0.264	2.819
65 years or older	-0.028	0.630	0.964	0.972	0.283	3.341
18–24 years	.	.	.	.	.	.
**Sex**						
Male	0.187	0.170	0.271	1.205	0.864	1.680
Female	.	.	.	.	.	.
**Country**						
Germany	-0.219	0.307	0.476	0.804	0.441	1.466
France	-0.436	0.279	0.118	0.647	0.374	1.117
USA	-0.127	0.322	0.694	0.881	0.468	1.657
Canada	-0.235	0.267	0.379	0.791	0.468	1.335
UK	.	.	.	.	.	.
**Dog’s Age**						
5–8 years	0.090	0.219	0.680	1.094	0.713	1.680
8–11 years	-0.090	0.218	0.681	0.914	0.596	1.403
11 years or older	.	.	.	.	.	.
**Is this your first dog?**						
Yes	-1.087	0.535	0.042	0.337	0.118	0.962
No	.	.	.	.	.	.
**Which one of the following best reflects your own dietary routine (select all that apply)?**						
Selected 1 option	0.079	0.202	0.695	1.083	0.728	1.609
Selected 2–4 options	0.475	0.255	0.063	1.608	0.975	2.652
Selected 5 or more options	0.532	0.429	0.214	1.702	0.735	3.943
Selected no options	.	.	.	.	.	.
**When choosing a pet food, I look for…**						
Weight control	-0.652	0.198	0.001	0.521	0.354	0.768
**I restrict my dogs food intake to control weight.**						
True	-0.490	0.192	0.011	0.613	0.420	0.893
False	.	.	.	.	.	.
**I provide a fixed amount of food to my dog every day.**						
True	0.352	0.248	0.155	1.422	0.875	2.310
False	.	.	.	.	.	.
**Other food (dog treats, table scraps, fruits/veggies, other) items given on a daily basis.**						
One option selected	-0.519	0.192	0.007	0.595	0.409	0.867
Two or more options selected	-0.783	0.235	0.001	0.457	0.288	0.724
No options selected	.	.	.	.	.	.
**Has anyone (including your veterinarian) ever told you that your dog is overweight?**						
Yes	-3.405	0.180	<0.001	0.033	0.023	0.047
No	.	.	.	.	.	.
**In a typical week, how many days do you spend more than 15 minutes vigorously exercising?**						
More than 5 days/week	1.022	0.356	0.004	2.780	1.384	5.583
1–2 days/week	0.382	0.226	0.091	1.464	0.941	2.279
2–3 days/week	0.379	0.273	0.166	1.460	0.855	2.494
3–4 days/week	0.231	0.298	0.438	1.260	0.703	2.259
4–5 days/week	1.241	0.404	0.002	3.459	1.567	7.635
No days/week	.	.	.	.	.	.
**How much physical activity (including walks) does your dog get per day?**						
0–15 minutes	-0.790	0.381	0.038	0.454	0.215	0.956
15–30 minutes	-0.372	0.349	0.286	0.689	0.348	1.365
30–60 minutes	-0.247	0.339	0.467	0.781	0.402	1.519
60–90 minutes	0.154	0.375	0.682	1.166	0.559	2.431
More than 90 minutes	.	.	.	.	.	.
**What type of exercise does your dog typically get in a regular week?**						
Vigorous exercise (run, playing ball, swimming, agility)	0.612	0.174	<0.001	1.844	1.310	2.596
Moderate exercise (walk, hike, off leash walk, dog park)	.	.	.	.	.	.
Age 65 plus*USA	0.085	0.415	0.838	-	-	-
First dog*0–15 minutes daily dog exercise	2.036	0.798	0.011	-	-	-
First dog*15–30 minutes daily dog exercise	2.277	0.683	0.001	-	-	-
First dog*30–60 minutes daily dog exercise	2.432	0.667	<0.001	-	-	-
First dog*60–90 minutes daily dog exercise	1.010	0.717	0.159	-	-	-
First dog*More than 90 minutes daily dog exercise	.	.	.	.	.	.

^1^ Estimated multinomial logistic regression coefficient

^2^ Odds Ratio or exponentiation of the coefficient (β)

^3^ 95% Confidence Interval of the Odds Ratio

McFadden Pseudo R-Square = 0.40

Dependent variable categories, 1 = responded ‘yes’ to “Do you believe that you dog is an ideal body weight?”, 0 = responded ‘no’ to “Do you believe that you dog is an ideal body weight?”

## Discussion

The goal of the current survey was two-fold. First, to examine how a dog owner’s exercise routine influences their dog’s exercise routine, and second, to investigate which dietary and exercise variables influence a dog owner’s perception of their dog’s body weight. An owner’s exercise routine, but not an owner’s dietary routine, influence both their dog’s exercise routine and, in turn, their perception of their dog’s body weight. Owners who performed any amount of vigorous exercise were more likely to have a dog that performed vigorous exercise. In addition, owners that performed moderate exercise for more than 5 days per week were more likely to exercise their dogs for 60–90 or more than 90 minutes per day, although this was only slightly higher than those who performed moderate exercise for fewer days per week. In turn, owners who performed vigorous exercise for 4–5 or 5 or more days per week and owners who reported that their dog performed vigorous exercise were more likely to believe that their dog is an ideal body weight. It is important to note the potential ambiguity in the categories of the multi-select questions. For example, owners who exercise 2 days per week, could have selected either 1–2 days or 2–3 days. However, given that these questions were worded to be an estimate of the amount of exercise in a typical week and that respondents may not exercise the same amount each week, this was intended to be range so that the respondent could select what an average week of exercise might look like. In addition, the results of the survey suggest that the larger trends of vigorously exercising for any amount of time versus not exercising still hold and differences in 1–2 or 2–3 days of exercise are small and less important in terms of the overall picture.

To the authors’ knowledge, this is the first large scale, international survey to demonstrate a relationship between an owner’s exercise routine and their dog’s exercise routine. However, a survey conducted in Australia, demonstrated that a number of variables were predictive of an owner’s intent to exercise their dog, most notably, was their own value of exercise [18]. Furthermore, the stronger the owner’s intention to exercise their dog appropriately, the more exercise their dog actually received. Similarly, the results from the current survey suggest that dog owners in Germany place higher value on exercise in terms of their dog’s overall health and in turn, exercise their dog for longer periods of time and are less likely to have been told their dog is overweight and more likely to believe that their dog is an ideal body weight. In support of this, those who value the benefits of dog walking and those who enjoy dog walking partake in more dog walking per week compared to those who engage in dog walking due to guilt or because others say that they should [19]. Taken together, this suggests that an owner’s perception of the importance of exercise in their own life influences the amount of exercise their dog receives which influences their perception of their dog’s body weight.

In contrast, the survey in Australia, mentioned above, reported that an owner’s intent to feed their dog appropriately did not predict the owner’s actual feeding behaviour [18]. This highlights a disconnect between feeding and exercise behaviours and suggests that perhaps despite an owner’s best intentions, they may not be feeding their dog appropriately. This is supported by the notion that an owner’s love for their dog is often shown through the provision of food or treats [20] and that owners of obese dogs are more likely to give in to begging behaviours [10]. Similarly, in the current survey, owners who have been told their dog is overweight were more likely to report giving their dog two or more other food items on a daily basis and restricting their dog’s food intake to control weight. Not only is over-feeding treats and under-feeding kibble counter-intuitive, it may also be detrimental to the overall nutrition provided to the dog. It is important to note that this survey only considered respondents who feed a commercial kibble diet to their dog, so, if the owner is restricting a diet intended for maintenance or even one that is intended for weight control, there is the possibility of restricting essential nutrients, potentially leading to a nutritional deficiency [21, 22]. Together, these data may suggest that new approaches to weight management should be explored with a focus on responsibly feeding pet foods and other food items and more of an emphasis on increased exercise.

Furthermore, the results from multinomial linear regression suggest that owners who reported restricting their dog’s food to control weight, giving one or two or more other food items on a daily basis and selecting a weight control food for their dog were all less likely to believe that their dog is an ideal body weight. These findings suggest that an owner who believes their dog is not an ideal body weight may attempt to control that by restricting the dog’s food or using a weight control diet, but not changing the dog’s exercise routine, seeing as those who have been told their dog is overweight are more likely to exercise their dog for only 0–15 minutes per day. Furthermore, it can be assumed that selecting a weight control diet or restricting your dog’s food intake is a consequence of believing your dog is overweight. Among those who selected a weight control diet, there was no difference in the amount of exercise the dog received and among those who reported restricting their dog’s food intake, only a weak relationship existed between that and exercise. In the study done in Australia mentioned above, an owner’s motivation to comply with a veterinarian’s exercise recommendations were lower than their motivation to comply with a veterinarian’s feeding recommendations [18]. Seeing as changing a dog’s feeding routine requires little change in the owner’s routine, but changing a dog’s exercise routine often requires the owner to make changes to their own exercise routine, this makes sense. Although it can be difficult to make lifestyle changes, studies have demonstrated the beneficial effect of dog walking on both owner and dog in terms of weight loss and improved physical exercise, highlighting the importance of a One Health approach to weight loss [23, 24].

It is important to highlight that, in the current survey, the outcome variable used in the multinomial linear regression was, “do you believe you dog is an ideal body weight?” Dog’s body condition score was not confirmed and respondents were not asked specifically if they thought their dog was overweight. However, in many surveys that do ask for a BCS, only an extremely small number of dogs are underweight and those are most often younger dogs [11, 18, 25]. By eliminating dogs aged 0–5 years in the regression and using variables that were thought to be related to being overweight, we attempted to capture and address variables that are important to an owner’s perception of an overweight or obese dog. Additionally, believing your dog is an ideal body weight was negatively correlated with someone (including a veterinarian) telling you that your dog is overweight, therefore it may serve as proxy for a veterinarian diagnosing the dog as overweight. Furthermore, the goal of this survey was to examine which exercise and dietary habits contribute to an owner’s *perception* of their dog’s body weight, not to address any disconnect between what an owner believes is an ideal body weight versus what is actually overweight. Since we know that many owners inaccurately assess their dog’s body condition [14], the percentage of respondents who reported believing that their dog is an ideal body weight may be an overrepresentation of the actual frequency of ideal weight dogs. Future studies may attempt to correlate the amount of exercise a dog receives with an owner’s perception of their dog being an ideal body weight in comparison to their dog’s actual BCS.

When the variable, “has anyone (including a veterinarian) ever told you that your dog was overweight,” was added to the regression model in the current survey, the Pseudo R-square value increased by more than double. This suggests that someone else, including a veterinarian, telling you that your dog is overweight holds a lot of value. This is in agreement with a survey in Australia, in which owners ranked veterinary guidance first among six weight loss strategies [7]. Other strategies in order of most preferred to least preferred included; modify diet, veterinary weight loss product, increase exercise, visit an obesity clinic and eliminate treats [7]. Modifying the dog’s diet or using a veterinary weight loss product again supports the findings from the current survey and suggests that owners will sooner change their dog’s diet than increase exercise or eliminate treats. Although veterinarians reported that the main causes of obesity in their patients was attributed to diet, socioeconomic reasons and exercise, the highest ranked strategies that veterinarians reported using to address obesity was to reduce food, reduce treats and change the diet, not to change the exercise routine. Taken together, this highlights an avenue that should be addressed by veterinarians as they continue to diagnose, treat and prevent obesity, and that is, exercise.

Nearly every other survey discussed, thus far, reported that dogs who exercise less frequently or less intensely are more likely to be overweight [1, 10–12, 25], yet changing the dog’s exercise regimen is ranked as a less common option by both owners and veterinarians [7]. In order to treat and prevent obesity in dogs, a more holistic approach to changing both dietary and exercise routine needs to be made. It has been well established in both human and canine studies that when even a moderate amount of exercise is added to a controlled diet regimen, genomic changes to metabolism in both muscle and adipose tissue, as well as maintenance of lean body mass are observed compared to only implementing a controlled diet regimen [26–28]. Furthermore, it has long been established that exercise in general, not to lose weight, provides multiple psychological benefits, contributing to overall well-being [29–31]. This may mean that rather than simply providing owners with information, it may be necessary to improve their awareness, intentions, and perhaps even their perceptions, related to the importance of diet and exercise in terms of their dog’s body weight, welfare and overall health [18]. In terms of how veterinarians communicate with their clients, previous work reported that despite the majority of owners acknowledging that they had discussed body weight with their veterinarian, 39% of owners with overweight dogs still believed that their dog was an ideal body weight, despite the veterinarian scoring the dog as overweight [14]. This opens the door to another avenue of research investigating how an owner’s underlying beliefs about exercise and bodyweight translate into how they approach weight loss and dietary management under the guidance of their veterinarian.

In conclusion, the results from this survey highlight the need to incorporate exercise strategies into weight loss programs for dogs and encourage dog owner’s to exercise their dog as a preventative measure to avoid weight gain. In order to do this, more resources for veterinarians are needed to help implement exercise regimens in their patients. Owners who spent more time exercising were more likely to exercise their dog more and in turn, believe that their dog is an ideal body weight. Thus, owner attitudes towards exercise in their own life may be transferred to their attitudes toward the importance of exercise in terms of their dog’s overall heath, highlighting the importance of a One Health approach to owner and dog exercise habits and weight loss.
